# Modeling maternal cholesterol exposure reveals a reduction of neural progenitor proliferation using human cerebral organoids

**DOI:** 10.1093/lifemedi/lnac034

**Published:** 2022-08-26

**Authors:** Pan Fan, Yuanhao Wang, Kaiqin Lu, Yuan Hong, Min Xu, Xiao Han, Yan Liu

**Affiliations:** Institute for Stem Cell and Neural Regeneration, State Key Laboratory of Reproductive Medicine, School of Pharmacy, Nanjing Medical University, Nanjing 211166, China; Institute for Stem Cell and Neural Regeneration, State Key Laboratory of Reproductive Medicine, School of Pharmacy, Nanjing Medical University, Nanjing 211166, China; Institute for Stem Cell and Neural Regeneration, State Key Laboratory of Reproductive Medicine, School of Pharmacy, Nanjing Medical University, Nanjing 211166, China; Institute for Stem Cell and Neural Regeneration, State Key Laboratory of Reproductive Medicine, School of Pharmacy, Nanjing Medical University, Nanjing 211166, China; Institute for Stem Cell and Neural Regeneration, State Key Laboratory of Reproductive Medicine, School of Pharmacy, Nanjing Medical University, Nanjing 211166, China; Institute for Stem Cell and Neural Regeneration, State Key Laboratory of Reproductive Medicine, School of Pharmacy, Nanjing Medical University, Nanjing 211166, China; Institute for Stem Cell and Neural Regeneration, State Key Laboratory of Reproductive Medicine, School of Pharmacy, Nanjing Medical University, Nanjing 211166, China

**Keywords:** brain organoids, maternal high cholesterol, neurodevelopmental disorders

## Abstract

Maternal obesity raises the risk of high-cholesterol exposure for their offspring. Studies in cohorts and animal models report that maternal obesity could increase the risk of neurodevelopmental disorders in offspring including intellectual disabilities and autism spectrum disorders (ASDs). However, whether exposure to high cholesterol is responsible for brain developmental defects, as well as its underlying mechanism, is still unclear. Here, we constructed a cholesterol exposure model utilizing human pluripotent stem cell (hPSC)-derived cerebral organoids by exogenously adding cholesterol into the culture system. We observed enlargement of endosomes, decreased neural progenitor proliferation, and premature neural differentiation in brain organoids with the treatment of cholesterol. Moreover, in comparison with published transcriptome data, we found that our single-cell sequencing results showed a high correlation with ASD, indicating that high cholesterol during maternal might mediate the increased risk of ASD in the offspring. Our results reveal a reduction of neural progenitor proliferation in a cholesterol exposure model, which might be a promising indicator for prenatal diagnosis and offer a dynamic human model for maternal environment exposure.

## Introduction

The population of obesity among women is growing world widely [1, 2]. Maternal obesity before or during pregnancy has been reported to be implemented in the offspring’s brain development [3, 4], which may induce the occurrence of altered neurodevelopmental or mental disorders [5]. Cohort studies have displayed that the risks of ASD [6, 7], depression [8, 9], and schizophrenia [10] are increased in the offspring whose mother has obesity. However, the bridge between maternal obesity and offspring’s neurodevelopmental deficits is still unclear, mainly because human brain models for dynamic studies of toxicity exposure during early pregnancy is lacking.

Exploring the association between the human maternal environment and neurodevelopmental disorders (NDDs) in offspring is not easy [11]. Studies in animal models have been carried out to inquire about the impact of maternal obesity on offspring’s neurodevelopment [11–13]. Mice fed a 40%–60% fat diet could induce offspring’s extensive structural brain changes and a high expression of genes associated with ASD [11]. Moreover, a high-fat diet might alter offspring neurodevelopment and result in anxiety as adults in rats [13]. Though rodent studies suggest that maternal high cholesterol could increase the risk of NDDs in offspring, the underlying mechanisms have not been elucidated. Nevertheless, there are species differences in placental anatomy and molecular function between humans and rodents. The rodents have a second placenta called the inverted yolk sac placenta, which is completely absent in humans [14, 15]. Thus, rodent models cannot fully mimic the human placenta’s precise transfer process [16]. Moreover, the human placenta shows gene clusters and estrogen synthesis in comparison to the rodent placenta [17–19]. These structure and genetic differences in the placenta between humans and rodents limit the application of rodent models in the studies of environmental exposure during pregnancy.

hPSCs and brain organoid techniques have offered an opportunity to assess developmental neural toxicants. For example, brain organoids with prenatal cocaine exposure showed altered neocorticogenesis and decreased proliferation of progenitor cells [20]. Methamphetamine (METH) exposure to brain organoids indicated METH regulates neural stem cell proliferation, differentiation, and cell death by neuroinflammatory [21]. These studies indicate that brain organoids have been provided a human model system to investigate the maternal environment exposure to NDDs in offspring.

Obesity causes cholesterol concentration to increase in the peripheral blood. In adults, cholesterol in plasma is unable to pass the blood–brain barrier, thus cholesterol in neurons is mainly synthesized by astrocytes in the brain [22]. While at the early embryonic development, the fetal blood–brain barrier is not yet established until 12 weeks of gestation [23, 24]. Maternal cholesterol could cross the blood–fetal barrier and enter the fetal brain [25–27]. And the cholesterol in the fetus is largely dependent on the maternal supply at the early developmental stages [26]. Notably, our previous studies evidenced that transcript levels of human brain organoids cultured for a month are similar to those of human gestation at 8–9 weeks [28]. Thus, brain organoids could recapitulate the early development stages of the human brain.

In this study, we found maternal cholesterol exposure affects the proliferation and differentiation of neural stem cells by exogenously adding cholesterol into the brain organoid culture system. Our results showed that the endogenous cholesterol levels in cerebral organoids are significantly increased after cholesterol treatment. And the cholesterol-treated (cho-treated) cerebral organoids presented enlarged and aggregated endosomes in the ventricular zone (VZ)-like regions, reduced neural progenitor proliferation, and premature neural differentiation. Our study provides a dynamic human model to study the maternal potential toxicity exposure at early gestation.

## Results

### Cholesterol treatment induces enlargement of endosomes in the VZ-like region

**Figure 1.**
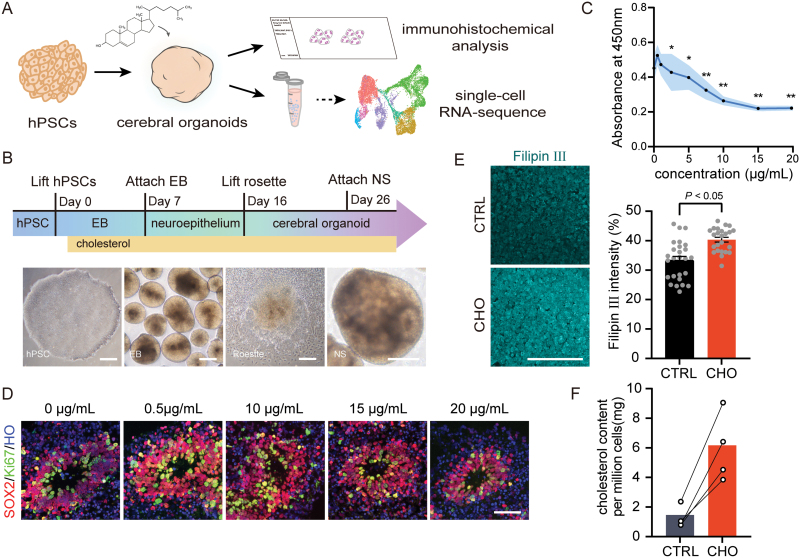
The establishment of cho-treated organoid models. (A) Schematic illustrating that exogenous cholesterol was added during the whole process of cortical organoid differentiation to simulate high-cholesterol developmental environment. At day 30, slice immunofluorescence staining analysis and single-cell transcriptome sequencing analysis were performed. (B) Schematic diagram of hPSC differentiation to cerebral organoids. Scale bar = 100 μm. (C) Statistical line chart of neural stem cell proliferation ability under different cholesterol concentrations detected by CCK8. *P < 0.05, **P < 0.01. (D) Immunofluorescence staining for the neural progenitor marker SOX2 and newborn neurons marker DCX in D30 cerebral organoids cultured at different cholesterol concentrations. Scale bar = 100 μm. (E) Filipin Ⅲ staining of D30 cerebral organoids slices (left). Scale bar = 100 μm. Statistics graph of filipin Ⅲ fluorescence intensity (right). *P* ＜ 0.05. (F) Quantification of cholesterol content per million cells in CTRL and CHO groups.

To establish an *in vitro* model that mimics the cholesterol environment during early pregnancy, we generated cerebral organoids based on our previously established cerebral organoids differentiation protocol and supplied exogenous cholesterol into the culture medium continuously, (Figs. 1B and S1A) [28]. As cholesterol is insoluble in water, we used methyl-beta-cyclodextrin/cholesterol complex (MβCD-cholesterol) as the primary cholesterol supplement reagent, which could directly deliver cholesterol to the membrane of cultured cells [29, 30], and increased the intracellular cholesterol levels [31]. Our previous research has reported that the transcriptome level of day 30 (D30) cerebral organoids was similar to the human fetus brain at 8–9 weeks of gestation [28], which is a critical stage of embryonic development. Thus, we perform an assay on D30 organoids (Fig. 1A). First, we set a gradient of concentration based on the concentration of cholesterol in cerebrospinal fluid and amniotic fluid reported in the fetus [26, 32, 33]. The viability of neural stem cells at each cholesterol concentration was examined using the cell counting kit-8 at D30 (Fig. 1C and 1D). In addition, we observed that brain organoids treated with cholesterol at the concentration of 20 μg/mL exhibited a dark and hairy-sphered appearance, indicating that the concentration of 20 μg/mL cholesterol may be toxic to the growth of the brain organoids (Fig. S1B). Combining the results of cell viability curves and histological assays, we determined to treat cerebral organoids with cholesterol at the concentration of 15 μg/mL throughout our experiments. Then, we used filipin Ⅲ, a typical reagent for visualizing the cellular distribution of free cholesterol [34], and a cholesterol quantitation kit to detect the level of cholesterol in the brain organoids (Fig. 1E and 1F). The results showed a significant increase in the concentration of cholesterol in the organoid with exogenous addition of cholesterol, which indicated that we have established a cerebral organoid model of cholesterol exposure.

**Figure 2.**
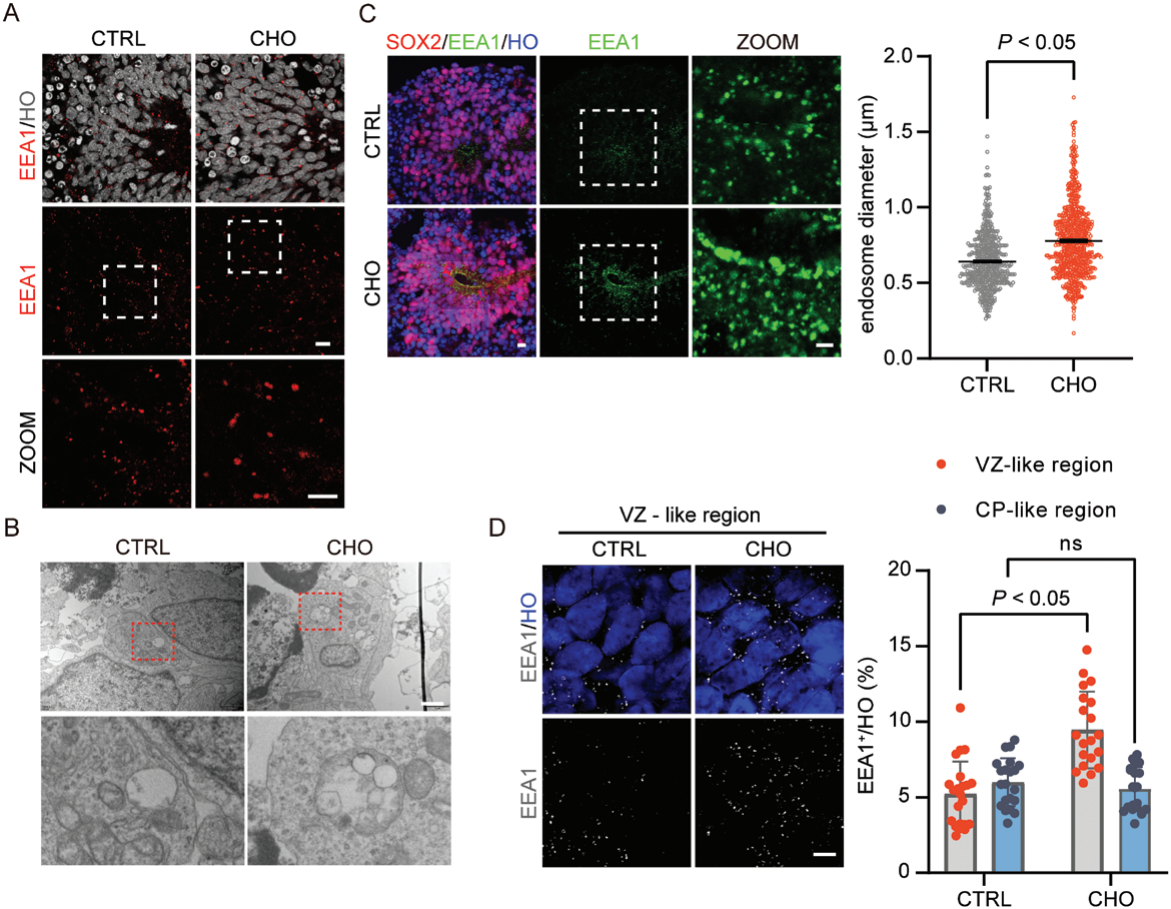
Cholesterol treatment-induced enlargement of endosomes in VZ-like region of brain organoids. (A) Immunofluorescence staining for endosomal marker EEA1 of D30 cerebral organoids slices. Scale bar = 100 μm. (B) Electron microscopy of endosomes in D30 cerebral organoids. Scale bar = 1 μm. (C) Immunofluorescence staining for the neural progenitor marker SOX2 and endosomal marker EEA1 of D30 cerebral organoids slices more specifically (left). Scale bar = 100 μm. Quantification of endosome diameter in CTRL and CHO groups (right). *P* ＜ 0.05. (D) Super Resolution Microscope (N-SIM) of immunofluorescence staining for EEA1 in VZ-like region of D30 cerebral organoids (left). Scale bar = 5 μm. Quantification of EEA1 numbers in VZ-like and CP-like regions (right). *P* ＜ 0.05 for CTRL VZ-like region vs CHO VZ-like region, ns for CTRL CP-like region vs CHO CP-like region.

Previous studies demonstrated that cholesterol exposure could lead to endosomal enlargement in neurons, known as endosomal swelling [35, 36]. Then, we measured the size of endosomes in cho-treated cerebral organoids at D30. Notably, we found an increased size of endosomes in cho-treated brain organoids vs the controls (Fig. 2A and 2B). Since the swelling of endosomes is an indication of defected maturation, thus the increased size of endosomes suggested the maturation process of possible endosomes might be impaired [37–40]. In addition, we found that the size of lysosomes did not alter significantly (Fig. S1C), indicating that the lysosomal pathway was not affected. Moreover, we found that the endosomal swelling phenotype was more pronounced in the VZ-like region (Fig. 2C), and the number of endosomes in RGCs was also increased (Fig. 2D). These outcomes suggest that cholesterol exposure might affect RGC development.

### Single-cell RNA-Seq reveals altered neural progenitor proliferation and cholesterol metabolism in cholesterol exposure organoids

**Figure 3.**
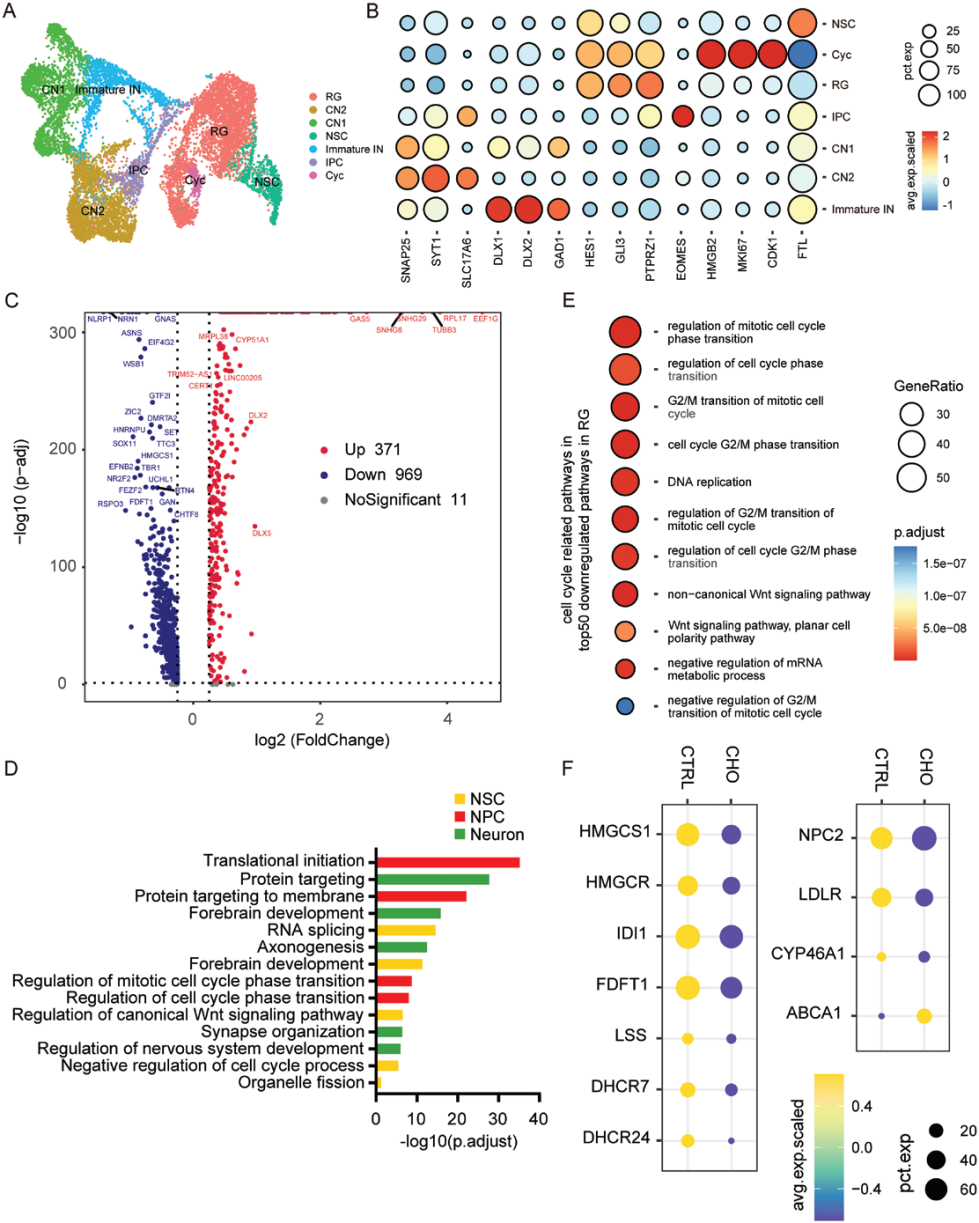
Analysis of single-cell RNA-seq revealed altered neural precursor proliferation and abnormal cholesterol metabolism. (A) UMAP plot showing the cell types detected in CTRL and CHO samples. RG, radial glial cells; CN2, CN1, cortical neuron; NSC, neural stem cells; Immature IN, immature inhibitory neurons; IPC, intermediate progenitor cells; Cyc, cycling cells. (B) Dot plot showing the expression level and percentage of representative marker genes across seven main cell types. (C) Volcano plot showing DEGs between high-cholesterol cultured organoids and control organoids. (D) GO analysis of DEGs across all clusters in biological processes. (E) Dot plot showing GO enrichment analysis of DEGs between high-cholesterol cultured organoids and control organoids in cell cycle-related pathways. (F) Single cell RNA sequence data showing the expression of cholesterol biosynthesis and metabolism-related genes.

To further investigate the effect of cholesterol exposure on neurodevelopment, we performed a single-cell RNA sequence (scRNA-seq). The unbiased clustering analysis defined seven cell clusters and was annotated according to the expression of known cell-type markers (Fig. 3A and 3B). We extracted differentially expressed genes (DEGs) through the following criteria: if the average log_2_FC > 0.25, the genes were assigned as up-regulated; If the average log_2_FC < −0.25, the genes were assigned as down-regulated. Transcriptome data revealed significant changes in cholesterol exposure organoids, with 371 up-regulated and 969 down-regulated DEGs (Fig. 3C, Table S1). Gene ontology (GO) analysis displayed that the DEGs in each cluster were enriched in biological processes, including translational initiation, forebrain development, and regulation of mitotic cell cycle phase transition (Figs. 3D, S1D and Table S2). Then, we found a higher enrichment of cell cycle-related pathways in RGCs, such as regulation of G2/M transition of the mitotic cell cycle (Fig. 3E, Table S3). These results suggest the proliferation of neural progenitor cells decreased in the cholesterol exposure group. Moreover, we uncovered that the expression of genes encoding cholesterol biosynthetic enzymes were up-regulated in the cho-treated group, such as HMGCS1, HMGCR, IDI1, FDFT1, LSS, DHCR7, and DHCR24, indicating that the biosynthesis of cholesterol in cho-treated brain organoids was down-regulated. Whereas the expression of genes encoding cholesterol efflux proteins such as ABCA1 was up-regulated, the gene encoding low-density lipoprotein receptor and CYP46A1, which converts cholesterol to 24S-hydroxycholesterol, were down-regulated in the cho-treated group [41, 42]. Also, the expression of NPC2, which encodes the NPC2 protein transporting intracellular cholesterol was down-regulated [43] (Fig. 3F). These changes indicate the sterol metabolic process was disrupted. The qPCR results also confirmed the same changes in cho-treated brain organoids as in scRNA-seq (Fig. S1E). The alterations in these genes indicated that cholesterol synthesis and metabolism were altered occurred in cho-treated brain organoids.

In addition, we also found alterations in the expression of endosome-related genes in brain organoids with cholesterol exposure. The process of endosome maturation includes a conversion from Rab5 to Rab7 [44, 45]. RAB5A and RAB7A are genes specifically expressed in early endosomes and late endosomes, respectively, and their dysregulation suggests abnormalities in the maturation process from early to late endosomes (Fig. S1E and S1F). This result is consistent with our earlier observation of swelling of the endosomes.

Taken together, our data suggest that cell proliferation of neural progenitors and cholesterol metabolic processes are altered in cho-treated cerebral organoids, which may result in abnormal neurodevelopment.

### The proliferation of neural progenitor cells was reduced in cholesterol exposure organoids

**Figure 4.**
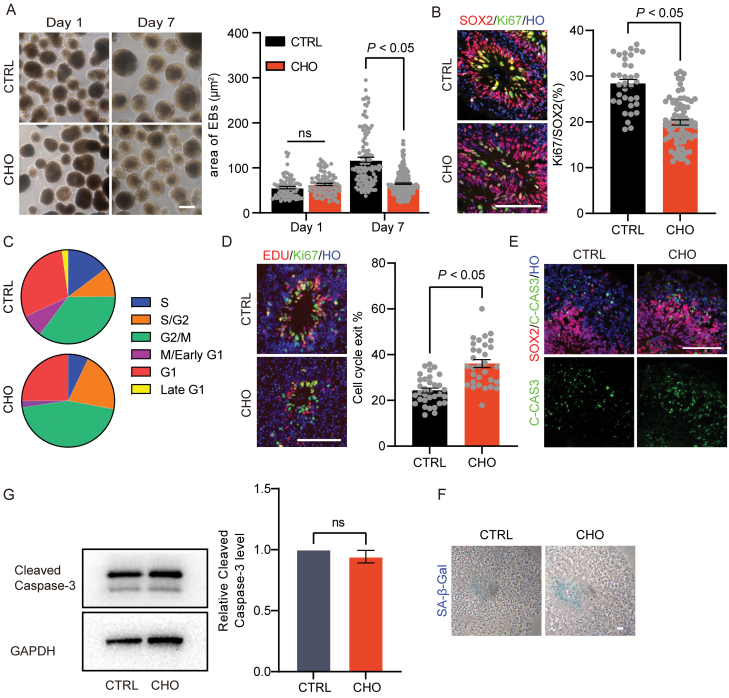
Reduced proliferation of neural precursor cells in cho-treated brain organoids. (A) Bright field pictures of EBs on day 1 and day 7 in high cholesterol and control group (left). Scale bar = 100 μm. Quantification of EB area on day 1 and day 7 in high cholesterol and control group (right). Ns for CTRL vs CHO group on day 1, *P* < 0.05 for CTRL vs CHO group on day 7. (B) Immunostaining for neural progenitor marker SOX2 and cell proliferation marker Ki67 in VZ-like region (left). Scale bar = 100 μm. Proportion of Ki67^ + ^cells of SOX2^+^ cells in CTRL and CHO groups (right). *P* < 0.05. (C) Pie chart showing the proportion of proliferating cells in cell cycle using the ccAF tool. (D) Representative images of EDU staining for CTRL and CHO groups (left). Scale bar = 100 μm. The percentage of cells exiting cell cycle in CTRL and CHO groups (right). *P* < 0.05. (E) Immunostaining for cell apoptotic marker C-CAS3. Scale bar = 100 μm. (F) SA-β-GaI staining for CTRL and CHO D30 cerebral organoids. (G) Western blotting analysis and quantification of Cleaved Caspase-3 expression in CTRL and CHO D30 cerebral organoids.

To confirm the alteration of the proliferation of neural progenitor cells in cho-treated cerebral organoids, we collected D30 brain organoids for cellular phenotype investigation. Initially, we observed a similar size of organoids between the cho-treated group and the controls at the beginning of differentiation (D1). While on D7, the size of cerebral organoids cultured in a cholesterol exposure environment was significantly reduced than that in the control group (Fig. 4A), indicating that cholesterol exposure may affect the proliferation of neural progenitor cells. Subsequently, we counted the ratio of ki67^+^ cells in the VZ-like region in D30 brain organoids and found that the percentage of Ki67 in the VZ-like region was significantly lower than in the cho-treated group (Fig. 4B). Moreover, we used the ccAF classifier to classify the cell cycle phase of the cells in neuroepithelial-derived stem and progenitor cell populations [46]. We found that the percentage of cells in the S-phase in the cho-treated group was remarkably decreased than controls (Fig. 4C). These results suggest that cholesterol exposure might lead to decreased proliferation of neural progenitor cells.

Next, we labeled D30 organoids for 1 h with 5-ethynyl-2ʹ-deoxyuridine (EDU), followed by chasing for 24 h, and then performed immunofluorescence to analyze cell cycle exit. The ratio of the total number of EDU^+^ KI67^−^ to the total number of EDU^+^ cells could be taken as the cell cycle exit index [47]. The cell cycle exit index of cho-treated organoids was significantly higher than the control (Fig. 4D). Furthermore, we performed cleaved caspase 3 and senescence-associated beta-galactosidase (SA-β-gal) assay to determine whether there was an elevation in apoptosis and senescence in the organoids with cholesterol exposure [48]. The treatment of cholesterol did not cause either the increased apoptosis or senescence in the cells, which indicates the decreased size of cho-treated organoids was mainly caused by the defected proliferation of neural progenitor cells, not cell apoptosis or senescence (Fig. 4E and 4F). The above outcomes suggest that cholesterol exposure could lead to an increased number of neural progenitor cells exiting the cell cycle, which could result in decreased numbers of neural progenitor cells.

### Cholesterol exposure-induced premature differentiation of neural progenitor cells

The cerebral brain organoid is made up of VZ-like region and cortical plate-like (CP-like) region. Cells in the VZ-like region are mainly neural stem cells and radial glial cells, which could generate newborn neurons, and then migrate to the outer region [49]. In the outer region of VZ-like region, there are mainly CTIP2^+^ and TBR1^+^ neurons, suggesting this region could be considered a CP-like fate [50].

**Figure 5.**
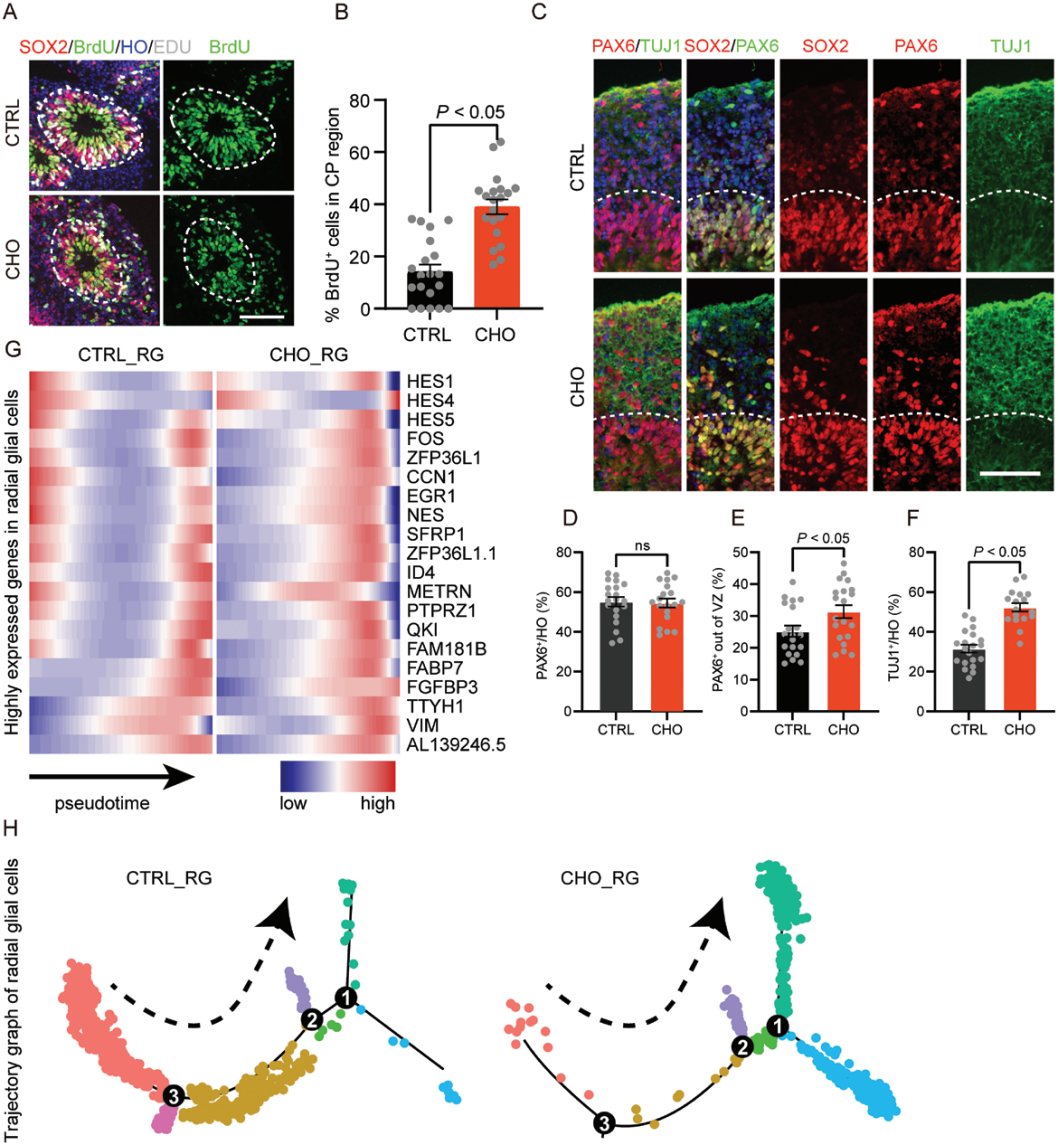
Cholesterol treatment induced neural precursor cells’ premature differentiation. (A) BrdU and EDU staining for CTRL and CHO D30 cerebral organoids. Scale bar = 100 μm. (B) The proportion of BrdU^+^ cells in the CP region. *P <* 0.05. (C) Immunofluorescence staining for neural progenitor cells marker PAX6, SOX2, and neuron marker TUJ1 in CTRL and CHO D30 cerebral organoids slices. Scale bar = 100 μm. (D) The proportion of PAX6^+^ cells in CTRL and CHO groups. (E) The proportion of PAX6^+^ cells out of VZ region in CTRL and CHO groups. *P* < 0.05. (F) The proportion of TUJ1^+^ cells in CTRL and CHO groups. *P* < 0.05. (G) The heat map shows the pseudotime of the highly expressed genes in radial glial cells. (H) The pseudotime trajectory map shows the trajectory graph of radial glial cells.

We labeled the D30 cerebral organoids with EDU for 1 h and then continuously labeled them with bromodeoxyuridine (BrdU) for 24 h. We found more BrdU-positive cells positioned in the CP-like region in the brain organoids in the cho-treated group in comparison with the controls (Fig. 5A and 5B). This suggests that more neural progenitor cells underwent asymmetric division to produce neurons during this period. Similarly, we also discovered more PAX6-positive cells outside the VZ-like region and a significantly higher proportion of TUJ1-positive neurons in cho-treated brain organoids (Fig. 5C–F). In addition, we utilized the R package monocle2 to perform pseudotime analysis of RGCs. The pseudotime trajectory map showed that the RGCs were mainly occurring at the later pseudotime in the cerebral organoids with cholesterol exposure, and the genes highly expressed in the RGCs such as CCN1, NES, VIM, et al. were also later estimated at time point (Fig. 5G and 5H). These results further confirm that cholesterol exposure could lead to premature differentiation of neural progenitor cells.

### Cholesterol exposure induced an autism-like transcriptional alteration in brain organoids

**Figure 6.**
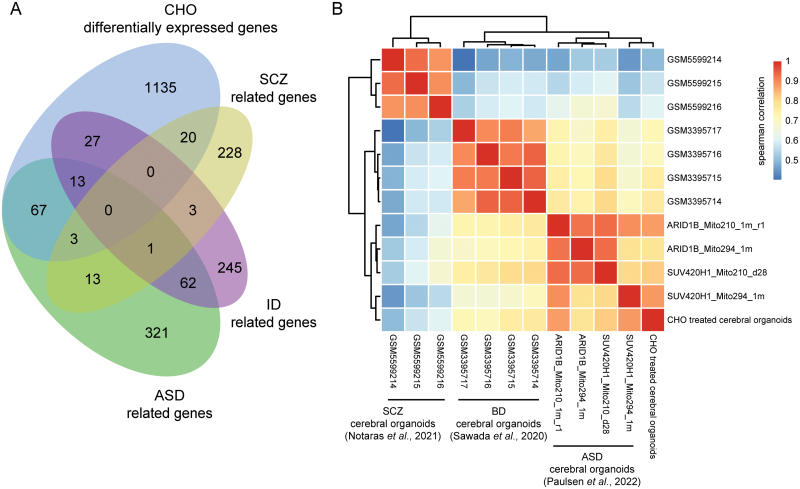
Cho-treated brain organoids showed similar gene expression profiles to ASD brain organoids. (A) The Venn diagram showed the DEGs between cho-treated and control brain organoids and genes associated with ASD, schizophrenia, and ID. (B) The heatmap showed the correlation ship in transcriptional profiles of cho-treated brain organoids and diseased brain organoids.

A maternal high-cholesterol environment could increase the risk of NDDs in offspring, including intellectual disabilities (ID), ASD, and other neuropsychiatric disorders [11, 13]. We compared the DEGs between cho-treated and control brain organoids with genes associated with ASD, schizophrenia, and ID [51]. The results showed that the differential genes had the most overlap with ASD-related genes (Fig. 6A). Furthermore, we correlated the scRNA-seq data of cho-treated brain organoids with published scRNA-seq data by using diseased brain organoids [52–54]. The transcriptional profiles of cho-treated organoids exhibited similarities to those of organoids derived from autism patients (Fig. 6B). These results indicated that the cho-treated brain organoids might partially recapitulate autism at transcriptional levels.

## Discussion

The increasing prevalence of obesity before or during pregnancy has been gradually widely concern, not only for mothers but also for their offspring’s health. Numerous studies, including in cohort and animal models, reveal that maternal obesity raises the risks of ASD, depression, and schizophrenia in their offspring. Studies in animal models have elucidated the convincing effect of maternal obesity on offspring’s cortical development. However, whether the information generated from animal assays could fully apply to human beings is still ambiguous. Human-derived cells, containing hPSCs and the derivations, could well compensate for the limitations of species differences in animal models. Hence, cerebral organoids might be an elegant human-derived model for investigating the mechanisms of neural developmental disorders caused by environmental exposure during pregnancy. In our study, we established a cholesterol exposure cerebral organoid for partially mimicking the high cholesterol of maternal obesity during pregnancy. After adding cholesterol into the culture medium, we confirmed an increased concentration of cholesterol in the cerebral organoids using filipin Ⅲ and cholesterol quantitation assay.

**Figure 7.**
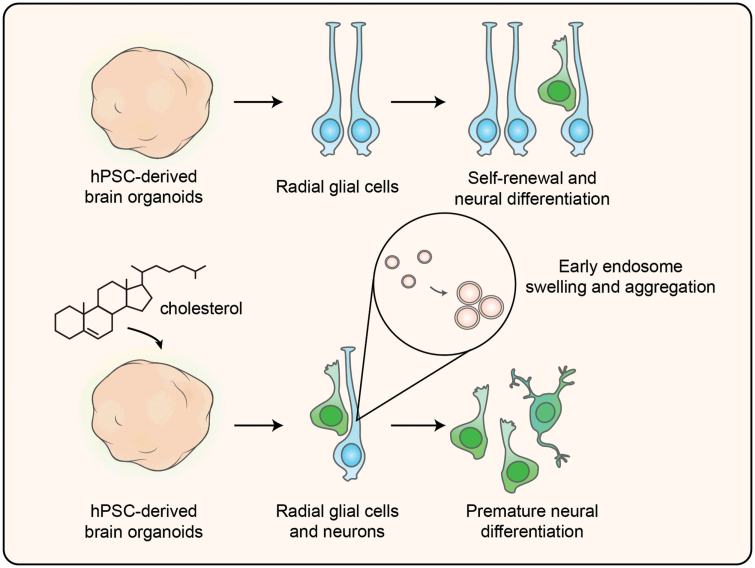
Graphical summary. Additional cholesterol exposure could cause premature neural differentiation of neural progenitor cells in human pluripotent stem cell-derived brain organoids, which might be one of the mechanisms underlying the increased risks of neurodevelopmental disorders in the offspring of mothers with obesity. Additional cholesterol caused endosome swelling and aggregation in the VZ-like region in brain organoids. The proliferation of neural progenitor cells was reduced in cholesterol exposure brain organoids. Cholesterol exposure-induced premature differentiation of neural progenitor cells in brain organoids.

Fetal brain development is precise and sensitive progress, which could be affected by multiple factors, such as environmental exposures [6, 8–10]. Before 12 weeks of gestation, the fetal blood–brain barrier is not established [23, 24], so cortical development is more susceptible to external interference. Owing to the dependency on the maternal supply of cholesterol at the early developmental stages, cholesterol could cross the blood–fetal barrier and enter the circulation of the fetus, which is served as a potential environmental exposure for cortical development. Studies for NDDs reported that environmental factors are involved in abnormal cortical development such as ASD, ID, and other neuropsychiatric disorders [11, 13]. Here, we discovered that cho-treated cerebral organoids showed a reduction of neural progenitor proliferation and premature neural differentiation (Fig. 7).

Previous studies have discovered that lipid metabolism can influence proliferation and neurogenesis. Cholesterol, as the major lipid in the brain, also plays an important role in neural proliferation. For example, the ablation of squalene synthase, one of the key enzymes in cholesterol biosynthesis, could cause the massive apoptosis of newborn neurons [55]. The cholesterol in the brain is mainly metabolized by CYP46A1 into 24-hydroxycholesterol (24-OHC). The 24-OHC has been proved to have cytotoxic effects on neuronal cells [56]. Our data showed that additional cholesterol treatment could upregulate the mRNA level of CYP46A1. Hence, we can anticipate that cholesterol metabolite 24-OHC might be increased, and the increased 24-OHC could induce the premature differentiation of radial glial cells. Besides, oxysterols could also take part in cell proliferation and differentiation [57]. Studies have shown that some oxysterols could inhibit cancer cell proliferation [58]. And other studies also revealed some oxysterols could stimulate MSCs differentiation [59]. Elevated oxysterols levels in brain organoids treated with additional cholesterol could trigger premature neuronal differentiation in a similar manner.

As known, ASD is a typical neurodevelopmental disease, which might be caused by environmental exposure. Notably, in comparison with published transcriptome data on ASD, we found that our data were highly correlated with ASD, which implies that maternal high cholesterol might be a candidate environmental risk factor for ASD.

## Research limitations

The *in vitro* concentrations of cholesterol used in our study may not faithfully represent the *in vivo* concentration during fetal brain development since the early differentiated organoids are sensitive to the high concentration of cholesterol (20 μg/mL) and tend to be unhealthy. Nevertheless, further studies are required. Taken together, we evidenced a decreased neural progenitor proliferation and premature neural stem cell differentiation using brain organoids under cholesterol exposure, which might be a promising indicator for prenatal diagnosis reverent to ASD. The cho-treated brain organoids provided a powerful model for studying environmental exposures in early pregnancy.

## Materials and methods

### hPSCs culture

hPSCs including H9 (WiCell Agreement No. 16-W0060), and IMR90-4 (WiCell Agreement No. 17-W0063) were maintained on plates precoated by vitronectin in Essential 8 medium (Life Technologies) at 37°C, 5% CO_2_ atmosphere. Half of the culture medium was changed every single day until the cells were full of about 75% of the well. To passage, cells were treated with Gentle Cell Dissociation Reagent (STEMCELL Technologies).

### Generation of forebrain organoids

To generate forebrain organoids, dispase (Life Technologies) was used to detach the hPSCs clones from the plate. Then the cells were resuspended and transferred into a flask with the medium of 50% E8 and 50% neural induction medium (NIM; DMEM/F12, 1% N_2_, and 1% NEAAs, Life Technologies) to form the embryonic bodies (EBs). The medium was removed and changed to NIM with DMH1 (Tocris) and SB (Tocris). The medium was renewed every day. The EBs were attached to the plates in the medium of NIM containing 10% FBS (Life Technologies) on day 7, and the medium was replaced with NIM after 10 h. The attached EBs were maintained with NIM and blown off on day 16. Then the tissues were transferred to a flask with a medium of NIM consisting of 2% B27 to form forebrain organoids. Cholesterol (MβCD-cholesterol, Sigma) was added to the medium from day 1 to day 30. Brain organoids were collected on day 30 to do further analysis.

### Organoids immunostaining and morphological analysis

The tissues were fixed with 4% paraformaldehyde (Sigma) and dehydrated by sucrose and embedded with O.C.T (SAKURA), then cut into 10-μm slices using a Leica Cryostat (CM1950). For immunostaining, the slices were first washed with PBS for 5 min to remove the O.C.T. Then the PBS consisted of 5% donkey serum (Millipore) and 0.1% Triton X-100 (Bio-Link) was used to treat blocked and permeabilize the tissues for 1 h. Incubate the slices with the primary antibodies (see detailed list in Table S4) at 4°C overnight. On the second day, the primary antibody was removed, and the tissues were washed with PBS three times, 10 min per time. Then the secondary antibody (see detailed list in Table S4) was added and incubated with the tissues in dark for 1 h, the secondary antibody was washed with PBS three times, 10 min per time. Coverslips were mounted for fluorescence imaging.

All the images were captured by Nikon microscope (80i), Zeiss confocal laser microscopy (LSM 800), or Nikon ultra-high resolution microscope (TI-E + SIM). The images and data were analyzed with ImageJ and GraphPad.

### Western blot

The organoids were washed three times with precooled PBS, then blown by protein lysis solution (the mixture of RIPA lysis buffer and protease inhibitor Cocktail, Roche.) for 30 min on ice. The protein was quantified using a BCA assay kit (beyotime). For electrophoresis, protein samples were loaded on the SDS polyacrylamide gels (SurePage) and separated under 120 V for 1 h. Then the protein was transferred to polyvinylidene fluoride membranes under the condition of 300 mA, for 2 h. Block the membrane by 5% milk, for 1 h. Then incubated the membrane with the primary antibodies (listed in Table S4) overnight at 4°C. On the second day, wash the membrane with Tris-buffered saline Tween (TBST) five times, 10 min per time. Then incubate the membrane with the secondary antibodies (listed in Table S4) for 2 h at room temperature. After that, wash the membrane using TBST three times to prepare for detection. The Chemiluminescent HRP Substrate (Millipore) was used for the detection.

### Quantitative real-time PCR

The organoids were treated with a Trizol Kit (Thermo) to extract the RNA. The PrimeScripttm RT reagent kit (Takara) was used to reverse transcribed the extracted RNA to cDNA and then used for qPCR. The qPCR was conducted with LightCycler^®^ (Roche). The primers were listed in Table S5.

### EDU and BrdU labeling assay

For the EDU labeling experiment, the operation was performed according to the instructions of Click-iT EDU Imaging Kits (Invitrogen). Briefly, 10 μM EDU was added in the medium of organoids for 1 h at the culture condition. Then the EDU medium was replaced with a fresh NIM medium after the organoids were washed by DMEM/F12 for three times. Fresh medium was added then. The organoids were collected for immunostaining after 24 h.

For BrdU and EDU double-labeling experiment, 10 μM BrdU (beyotime) was added in the medium of organoids for 23 h under the culture condition. Remove the medium and wash the organoids with DMEM/F12 for three times. Then the organoids were collected after incubation with NIM consisting of 10 μM EDU for 1 h. Before immunostaining, the sliced tissues were incubated with 1.5 M HCl for 30 min at room temperature. The BrdU imaging assay was conducted at the same time as immunostaining, and the EDU imaging was performed after the immunostaining experiment.

### Transmission electron microscope samples preparation

D30 organoids were treated with 2.5% glutaraldehyde solution at 4°C overnight. Then the solution was removed and the organoids were washed three times with PBS, 15 min per time. Add 1% osmium tetroxide and stay for 2 h at 4°C. Wash the tissues twice with PBS, 5 min per time. Add 2% uranyl acetate aqueous solution and stay for 2 h. The tissues were dehydrated gradient with the solution of 50% acetone and 100% acetone, respectively. Embed the tissues overnight with the mixture of 100% acetone and EPON812 resin at a ratio of 1:1. Then the embedded tissues were sliced into 0.05 μm. The slices were treated with Uranium staining for 30 min, followed by lead staining for 10 min, then prepared for electron microscope observation.

### Single-cell sequencing and data analysis

About 10 organoids were randomly selected on day 30 to perform scRNA-seq. The organoids were washed by DPBS (Life Technologies) with 2% FBS and incubated with TrypLE (Life Technologies) for 40 min at 37°C. Then wash the tissues with 2% FBS three times. Gently blow and disperse the organoids into single cells, then collect the supernatant. The following processing procedures were in accordance with the 10 × Genomics standard procedure. Single Cell 3ʹ Library and Gel Bead Kit V3 (10 × Genomics) was used to construct the scRNA-seq libraries.

For single-cell transcriptome data analysis, Cell Ranger (version 3.1.0) was used to align Reads with the human reference genome hg38 and convert the data to Seurat objects for subsequent analysis after quality control. For downstream analysis using Seurat (version 4.0.4), the expression matrix was first screened as follows: nUMI ≥ 1200, nGene ≥ 1000, log10GenesPerUMI > 0.80, mitoRatio < 0.15. The scRNA-seq data integration was conducted following the Seurat standard workflow [60]. The transcriptomic profiles were visualized by UMAP. Differently expressed genes were obtained through Seurat Find Markers function with the default set. And if the average log_2_FC > 0.25, the genes were assigned as up-regulated. If the average log_2_FC < −0.25, the genes were assigned as down-regulated. Then the differently expressed genes were subjected to GO enrichment analysis using ClusterProfile (version 4.1.4) [61]. All the GO enrichment analyses were conducted using the “biological process” subontology of GO (GO: BP) following the standard workflow of ClusterProfile. Cell cycle analysis of proliferating cells was performed using the ccAF classifier on Python (version 3.8.5) [46]. The pseudotime analysis was conducted by Monocle 2 [62]. The top20 highly expressed genes in radial glial cells were selected to draw the pseudotime heatmap. For the correlation analysis, differently expressed genes obtained in this study were used as an input, then calculate the average expression of these genes in each disease-derived brain organoids and compare the correlation of the gene expression profiles. Pearson correlation coefficient was used in the analysis.

## Data availability

The cho-treated day 30 brain organoids scRNA-seq data has been deposited to the GEO database with accession number GSE206587. The control brain organoids scRNA-seq data was previously published in our lab’s work (sc_CONT_IMR90-4_D30, SRS8707766 [28]).

## Statistical analysis

The data were analyzed by Student’s *t*-test for comparison of two groups or ANOVA followed by Tukey’s multiple comparison test for comparison of multiple groups. Each study was performed with at least three independent biological replications. The data were expressed as the mean ± SEMs. *P* < 0.05 was considered to achieve statistical significance.

## Supplementary Material

lnac034_suppl_Supplementary_Figure_S1

lnac034_suppl_Supplementary_Table_S1-S5
