# Supplementary figures and images for: Modeling maternal cholesterol exposure reveals a reduction of neural progenitor proliferation using human cerebral organoids
Source: Life Med. 2022 Aug 26;2(2):lnac034. doi: 10.1093/lifemedi/lnac034 (PMC11749704; doi:10.1093/lifemedi/lnac034)

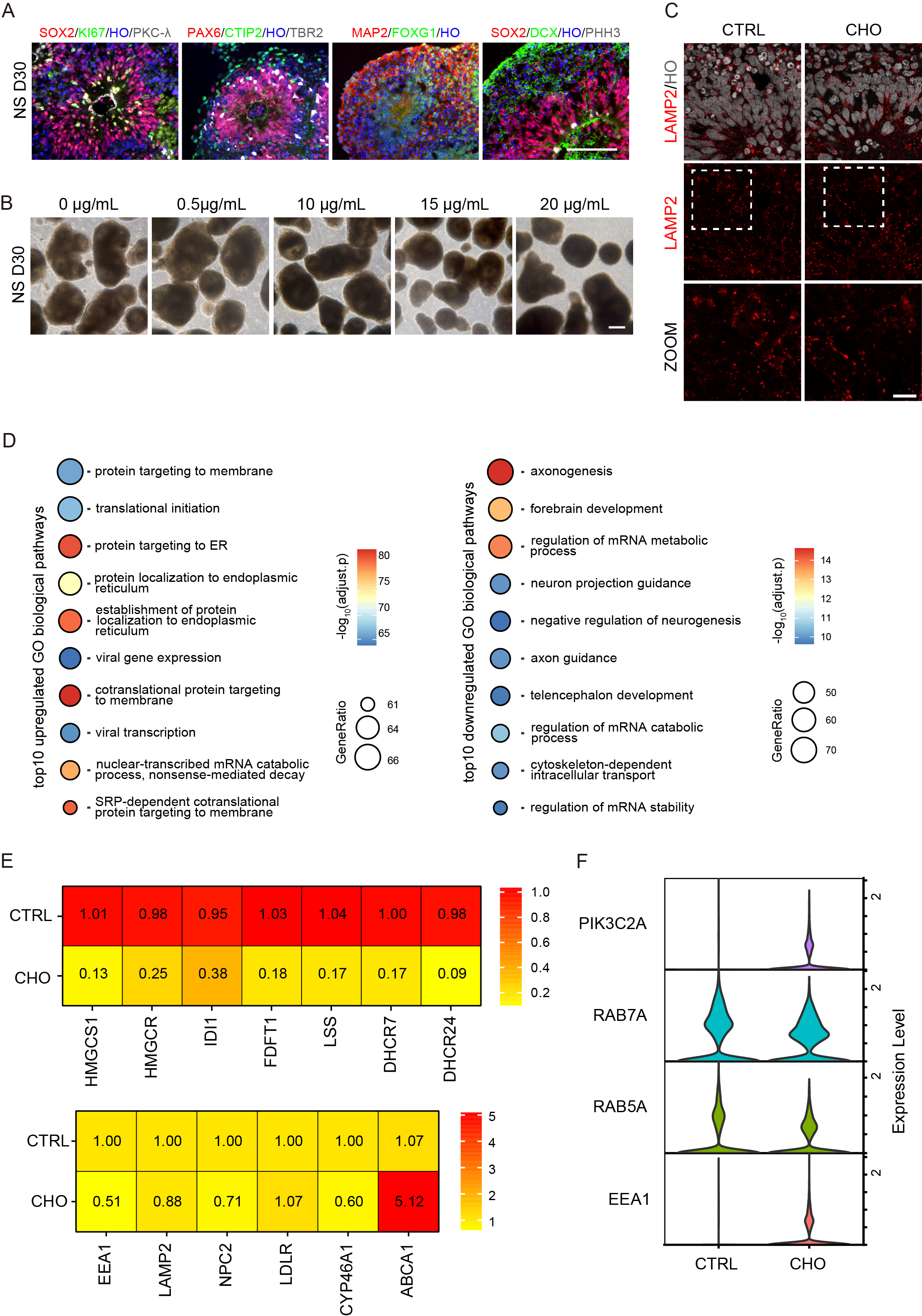

Supplement: lnac034_suppl_Supplementary_Figure_S1 [file lnac034_suppl_Supplementary_Figure_S1.bmp]
